# Assessing Chemical Diversity in *Psilotum nudum* (L.) Beauv., a Pantropical Whisk Fern That Has Lost Many of Its Fern-Like Characters

**DOI:** 10.3389/fpls.2019.00868

**Published:** 2019-07-09

**Authors:** Dunja Šamec, Verena Pierz, Narayanan Srividya, Matthias Wüst, B. Markus Lange

**Affiliations:** ^1^Institute of Biological Chemistry and M.J. Murdock Metabolomics Laboratory, Washington State University, Pullman, WA, United States; ^2^Ruđer Bošković Institute, Zagreb, Croatia; ^3^Chair of Bioanalytics, Institute of Nutritional and Food Sciences, University of Bonn, Bonn, Germany

**Keywords:** arylpyrone, biflavonoid, mass spectrometry, metabolomics, nuclear magnetic resonance, whisk fern

## Abstract

Members of the Psilotales (whisk ferns) have a unique anatomy, with conducting tissues but lacking true leaves and roots. Based on recent phyogenies, these features appear to represent a reduction from a more typical modern fern plant rather than the persistence of ancestral features. In this study, extracts of several Psilotum organs and tissues were analyzed by Gas Chromatography – Mass Spectrometry (GC-MS) and High Performance Liquid Chromatography – Quadrupole Time of Flight – Mass Spectrometry (HPLC-QTOF-MS). Some arylpyrones and biflavonoids had previously been reported to occur in Psilotum and these metabolite classes were found to be prominent constituents in the present study. Some of these were enriched and further characterized by Nuclear Magnetic Resonance (NMR) spectroscopy. HPLC-QTOF-MS and NMR data were searched against an updated Spektraris database (expanded by incorporating over 300 new arylpyrone and biflavonoid spectral records) to aid significantly with peak annotation. Principal Component Analysis (PCA) with combined GC-MS and HPLC-QTOF-MS data sets obtained with several Psilotum organs and tissues indicated a clear separation of the sample types. The principal component scores for below-ground rhizome samples corresponded to the vectors for carbohydrate monomers and dimers and small organic acids. Above-ground rhizome samples had principal component scores closer to the direction of vectors for arylpyrone glycosides and sucrose (which had high concentrations in above-and below-ground rhizomes). The unique position of brown synangia in a PCA plot correlated with the vector for biflavonoid glycosides. Principal component scores for green and yellow synangia correlated with the direction of vectors for arylpyrone glycosides and biflavonoid aglycones. Localization studies with cross sections of above-ground rhizomes, using Matrix-Assisted Laser Desorption/Ionization – Mass Spectrometry (MALDI-MS), provided evidence for a preferential accumulation of arylpyrone glycosides and biflavonoid aglycones in cells of the chlorenchyma. Our results indicate a differential localization of metabolites with potentially tissue-specific functions in defenses against biotic and abiotic stresses. The data are also a foundation for follow-up work to better understand chemical diversity in the Psilotales and other members of the fern lineage.

## Introduction

Free-sporing vascular plants encompass two distinct evolutionary lineages, the lycophytes and ferns, with the latter resolved as more closely related to seed plants (Kenrick and Crane, 1997; Pryer et al., 2001). Whisk ferns (order Psilotales), which comprise two genera (*Psilotum* and *Tmesipteris*) in the family Psilotaceae, have conducting tissues but no veins, and lack true leaves and roots. Water and mineral absorption occurs through underground, horizontally creeping rhizomes, sometimes in association with symbiotic fungi (mycorrhizae) (Ducket and Ligrone, 2005). Plants grow mostly as epiphytes (using other plants as physical support) in moist habitats. The stem-like aerial portion of rhizomes of members of the Psilotaceae is covered by an epidermis, followed inward by extensive cortical areas, a single-layered endodermis, and a thick-walled protostele that accommodates the water and nutrient-conducting tissues (Pittermann et al., 2011). The epidermal layer of the photosynthetic above-ground rhizomes contains stomata for gas exchange (Nilsen, 1995). In the genus *Psilotum*, above-ground rhizomes have many branches with scale-like appendages called enations. These structural outgrowths resemble miniature leaves but, unlike true leaves, have no internal vascular tissues. Above these enations, positioned laterally along the distal portions of aerial shoots, are spore-containing synangia, which result from the fusion of three adjacent sporangia (Renzaglia et al., 2001).

Because of its unusual anatomical characteristics, *P. nudum* was traditionally thought to be descended from the earliest vascular plants (Banks, 1975), and conflicting views regarding the placement of the Psilotales remained in the literature for decades. Recent phylogenies based on both morphological characters and extensive sequence data provided strong evidence that Psilotales, Ophioglossales (moonworts) and Marattiales (king ferns) – all eusporangiate ferns – form a monophyletic clade that is sister to leptosporangiate ferns, the largest group of living ferns (Doyle, 2018; Rothwell et al., 2018). The unique anatomy of extant Psilotales therefore appears to represent a reduction from a more typical modern fern plant rather than the persistence of ancestral features. While recent progress has been made with regard to resolving the classification of vascular plants, there is still a notable lack of knowledge regarding the phytochemical diversification associated with the adaptive radiation of ferns.

We selected *Psilotum nudum* (L.) Beauv. to evaluate chemical diversity in the fern lineage, as only limited knowledge exists on this topic. Psilotin and 3’-hydroxypsilotin are unusual C_11_ arylpyrone glycosides unique to the Psilotaceae (McInnes et al., 1965; Tse and Towers, 1967; Balza et al., 1985; Takahashi et al., 1990). Psilotic acid is a C6-C4 organic acid that is structurally related to the psilotin aglycone (psilotinin) (Shamsuddin et al., 1985). Prominent flavonoid glyosides in the Psilotaceae are O-glucosides of the biflavonoid, amentoflavone, and C- and O-glycosides of the flavone, apigenin (Cooper-Driver, 1977; Wallace and Markham, 1978; Markham, 1984). A survey across sixteen pteridophytes (ferns and fern allies), including *P. nudum*, concluded that the sterol composition is generally similar to that of spermatophytes (seed plants), with β-sitosterol, campesterol and stigmasterol as principal constituents (Chiu et al., 1988). *P. nudum* tissues were also demonstrated to contain representatives of several phytohormone classes (auxins, cytokinins and gibberellins) (Takahashi et al., 1984; Abul et al., 2010). In this pilot study, which is the beginning of efforts to chart out the most abundant classes of specialized metabolites in ferns, we demonstrate the utility of multi-platform analyses for capturing the unique chemical fingerprints of different *P. nudum* organs and tissues. In addition, we report the tissue-level localization of the most prominent arylpyrone glycoside and biflavonoid constituents.

## Materials and Methods

### Chemicals and Solvents

Solvents for extraction and chromatography were of the highest commercial grade and obtained from Sigma-Aldrich (St. Louis, MO, United States). Deuterated solvents for nuclear magnetic resonance (NMR) spectroscopy were obtained from Cambridge Isotope Laboratories Inc. (Andover, MA, United States), with details in Table 2. All authentic standards, reference materials (red phosphorus, α-cyano-4-hydroxycinnamic acid, 9-anthracenecarboxylic acid, sinapic acid and vanillic acid) and reagents (*N*-methyl-*N*-(trimethylsilyl)trifluoroacetamide) were generally purchased from Sigma-Aldrich (St. Louis, MO, United States); exceptions: 2,5-dihydroxybenzoic acid (TCI America, Portland, OR, United States) and leucine enkephalin (Waters, Milford, MA, United States).

### Plant Growth

*Psilotum nudum* (L.) P. Beauv. plants had been established from rhizomes roughly 6 years before the initiation of the experiments described here. A voucher specimen was deposited with the John G. Searle Herbarium of the Field Museum (Chicago, IL, United States). Plants were maintained in a greenhouse under ambient lighting, with supplemental lighting during winter months provided by high-intensity discharge lamps. The daily light integral varied from 15 to 25 mol m^-2^ d^-1^. Temperatures ranged between 22 and 27°C and the humidity was set to 70%. At the time of harvesting, *P. nudum* produced synangia that, based on color (green, yellow or brown), could be differentiated into three developmental stages (immature, mature, and senescent). Five biological replicates were harvested at the same time of day (11:00 AM, Pacific Daylight Savings Time) for the following organs: below-ground rhizome, above-ground rhizome (stem), and (separately) green, yellow and brown synangia. Samples were immediately frozen in liquid nitrogen, freeze-dried (aerial parts for 5 days, rhizomes for 7 days). Lyophilized material was homogenized to a fine powder under liquid nitrogen using mortar and pestle. Defined quantities of homogenate were weighed out, placed in a 2 ml microfuge tube, and stored as aliquots at –20°C until further use.

### Metabolite Extraction and Derivatization for Analysis by Gas Chromatography – Mass Spectrometry

Frozen tissue homogenate from each sample (15 ± 3 mg) was transferred to 8 ml glass tubes and overlaid with 700 μL methanol (containing myristic acid-d_27_ (CDN Isotopes, Quebec, Canada) as internal standard at 1.5 mg/ml) and 25 μL water. Tubes were capped tightly and heated in a water bath to 70°C for 15 min, centrifuged for 2 min at 3,500 × *g*, and supernatants transferred to new 8 ml glass vials. To each supernatant, 700 μL of water and 375 μL of chloroform were added and the contents of the tube mixed with a multi-tube vortexer (VWR Scientific, South Plainfield, NY, United States) for 15 min at a speed setting of 4. Extracts were centrifuged for 15 min at 3,500 × *g*, the upper aqueous phase was combined with the first methanol extract (henceforth referred to as aqueous methanol extract), and the lower organic phase was collected separately (chloroform extract). The two extracts were separately evaporated to dryness [Vacufuge Plus for aqueous methanol extract (Eppendorf, Hauppage, NY, United States); EZ-Bio Evaporator for chloroform extract (GeneVac LTD, Ipswich, United Kingdom)]. Dried samples were derivatized just-in-time by adding 10 μL of a 40 mg/ml solution of methoxyamine hydrochloride in pyridine and shaking gently at 30°C for 90 min, then adding 50 μL of *N*-methyl-*N*-(trimethylsilyl)trifluoroacetamide (MSTFA; Sigma-Aldrich, St. Louis, MO, United States) and shaking gently at 37°C for 30 min. Samples were allowed to cool to room temperature, and the extract was transferred to a glass insert, which was then placed in a 2 ml glass reaction vial.

### Gas Chromatography – Mass Spectrometry Analysis

Gas chromatography – mass spectrometry (GC–MS) was performed under the following conditions: injection volume: 1 μL (splitless mode); GC instrument: 6890N (Agilent Technologies, Santa Clara, CA, United States), GC; column: DB-5MS + DG (30 m × 0.25 mm × 0.25 μm; J&W Scientific, Santa Clara, CA, United States); inlet temperature: 250°C; temperature program: start at 60°C, ramp to 320°C at 3°C/min, hold for 10 min; retention time locking: myristic acid-d_27_ at 42.06 min at an inlet pressure of 10.65 psi; MS instrument: 5975 MSD (Agilent Technologies, Santa Clara, CA, United States); transfer line temperature: 250°C; electron ionization at 70 eV. Data analysis was performed using ChemStation, version E.02.00.493 (Agilent Technologies, Santa Clara, CA, United States). Custom spectral databases (specifying retention time, a quantification signal and three qualifier ions) were created using authentic standards from our in-house library for the identification of GC–MS peaks (Supplementary Table S1). Peaks generated by unidentified analytes were annotated based on community reporting guidelines (Bino et al., 2004; Fiehn et al., 2007). Raw data values were normalized for sample weight and signal intensity associated with the internal standard. Normalized data values were *z*-transformed (autoscaled) prior to statistical analyses.

### Metabolite Extraction for High Performance Liquid Chromatography – Quadrupole Time-of-Flight – Mass Spectrometry

Frozen tissue homogenate from each sample (30 ± 5 mg) was transferred to a 2 ml reaction tube and extracted with 1 ml of 80% aqueous methanol (containing 6 mg/L anthracene-9-carboxylic acid as internal standard) for 10 min [multi-tube vortexer (VWR Scientific, South Plainfield, NY, United States) at highest speed setting] and subsequent sonication for 20 min (ultrasonic bath at highest intensity setting, Fisher Scientific, Hampton, NY, United States). Following centrifugation for 10 min at 13,000 × *g*, supernatants were filtered through 0.22 μm polypropylene filter material and collected in plastic inserts for 2 ml reaction vials.

### High Performance Liquid Chromatography – Quadrupole Time-of-Flight – Mass Spectrometry Analysis

High Performance Liquid Chromatography – Quadrupole Time-of-Flight – Mass Spectrometry (HPLC–QTOF–MS) was performed under the following conditions: HPLC system: 1290 system (Agilent Technologies, Santa Clara, CA, United States) consisting of thermo-controlled autosampler (set to 4°C), binary pump (operated at 0.6 ml/min), isocratic pump [operated at 0.1 ml/min flow rate to introduce a reference mass solution containing 300 nM purine (exact mass 120.043596 g/mol) and 250 nM hexakis-(1H, 1H,3H-tetrafluoropropoxy)-phosphazine (exact mass 921.002522 g/mol) in acetonitrile/water (95:5; v/v)], thermo-controlled column compartment (set to 60°C), and diode array detector (scanning range 210–600 nm, resolution 1.2 nm); injection volume: 10 μl; Column: HD Zorbax SB-Aq (100 × 2.1 × mm; 1.8 μm pore size, Agilent Technologies, Santa Clara, CA, United States); Mobile phase: 0.1% (v/v) formic acid in water (solvent A) and 0.1% (v/v) formic acid in acetonitrile (solvent B). Gradient: 5% B at start; linear gradients to 10% B at 5 min, 20% B at 10 min, 80% B at 35 min, 95% B at 45 min; QTOF–MS instrument: 6530 series with electrospray ion source (Agilent Technologies, Santa Clara, CA, United States); polarity: positive; drying gas flow rate: 10 L/min; drying gas temperature: 325°C; nebulizer pressure: 2.4 bar; m/z range: 100–1,200 (high gain mode); scan rate: 1.4 scans/s for MS and 4 scans/s for MS/MS. Data analysis was performed using the MassHunter Workstation software package [B.07.00, Qualitative Analysis and B.06.00, Profinder, Agilent Technologies, Santa Clara, CA, United States). For each detected peak, molecular feature extraction (considering retention time (tolerance window 1.30 s) and high mass accuracy (m/z tolerance window 10 ppm)], deconvolution, and alignment across samples were performed using the recursive feature extraction algorithm (settings: threshold of 10,000 counts and peak spacing tolerance of 0.0025 m/z). Quasi-molecular ions and adducts were considered ([M+H]^+^, [M+Na]^+^, [M+K]^+^, [M+NH_4_]^+^), as were the corresponding dimers. The minimum absolute height required for feature extraction in the recursive step was set to 10,000 counts (sum of all peaks for a given molecular entity), which had to be fulfilled in at least three of five biological replicates. The global filter was limited to 2,000 results. Peak annotation was performed based on a combination of chromatographic, mass spectral (accurate mass and MS/MS fragmentation patterns), evaluation of the literature, and searches against spectral databases (Table 1). Peaks generated by unidentified analytes were annotated based on community reporting guidelines (Bino et al., 2004; Fiehn et al., 2007). MS/MS spectra for identified peaks were submitted to MassBank (Horai et al., 2010) to expand a widely used community spectral resource. Normalized data values for HPLC–QTOF–MS peaks were *z*-transformed (autoscaled) and combined with the normalized and *z*-transformed GC–MS data (Supplementary Table S2). The combined HPLC–QTOF–MS and GC–MS data set were processed by Principal Component Analysis (PCA) using the *R* statistical package^1^, for which the settings and outcomes are summarized in Supplementary Table S3.

**Table 1:** Annotation of HPLC-QTOF-MS peaks.

Accurate Mass – Time Tag	Monoisotopic Mass (Measured/Calculated)	Δppm	Molecular Formula	MS (ESI-Positive)	MS/MS(ESI-Positive) (Varying Collision Energies)	Annotation	References; Further Evidence
BML-LCMS18-4.66-368.1101	368.1101/368.1107	1.79	C17H20O9	[M+H]^+^ 369.1198 [M+Na]^+^ 391.0999 [M+K]^+^ 407.0696 [2M+Na]^+^ 759.2115 [2M+K]^+^ 775.1595	10 eV: 391.0999 (100), 123.0436 (80), 207.0649 (55), 189.0564 (30) 50 eV: 123.0438 (100), 189.0540 (35), 227.0002 (34), 98.9747 (26), 199.0080 (23), 110.9751 (19), 115.0537 (18), 171.0427 (17), 147.0451 (16), 231.0246 (15)	3’-Hydroxypsilotin	Balza et al., 1985; NMR (Table 2; F2)


BML-LCMS18-4.76-530.1636	530.1636/530.1636	0.00	C23H30O14	[M+Na]^+^ 553.1530	30 eV: 553.1504 (100), 207.1641 (11) 50 eV: 123.0433 (100), 173.0570 (48), 85.0281 (23), 189.0512 (29), 115.0534 (26), 203.0539 (17), 116.9908 (15), 147.0437 (14)	3’-Hydroxypsilotinin-di-O-hexoside	


BML-LCMS18-5.12-514.1688	514.1688/514.1686	0.08	C23H30O13	[M+H]^+^ 515.1751 [M+Na]^+^ 537.1591 [2M+Na]^+^ 1051.3232	30 eV: 537.1603 (100), 191.0746 (40), 391.0731 (15), 173.0627 (14), 107.0520 (7) 50 eV: 107.0538 (100), 173.0650 (87), 201.0111 (26), 229.0230 (20), 117.0743 (12), 145.0677 (12), 98.9808 (11)	Psilotinin-di-O-hexoside I	


BML-LCMS18-5.20-352.1168	352.1168/352.1158	2.79	C17H20O8	[M+H]^+^ 353.1250 [M+Na]^+^ 375.1075 [2M+Na]^+^ 727.2223	10 eV: 375.1075 (100), 173.0598 (70), 107.0487 (63), 191.0699 (45), 123.0434 50 eV: 107.0492 (100), 98.9753 (78), 173.0588 (66), 110.9858 (64), 183.0123 (44), 127.0544 (43), 167.0168 (42), 117.0706 (33), 201.0072 (31), 145.0635 (25), 229.0171 (23),	Psilotin	McInnes et al., 1965; Authentic Standard


BML-LCMS18-5.52-206.0568	206.0568/206.0580	0.12	C11H10O4	[M+H]^+^ 207.0652 [M+Na]^+^ 229.0475	30eV: 207.0677 (100), 189.0546 (81), 123.0443 (62) 50 EV: 123.0451 (100), 189.0566 (29), 173.0595 (27), 147.0451 (12)	3’-Hydroxypsilotinin	


BML-LCMS18-6.83-514.1686	514.1686/514.1686	0.00	C23H30O13	[M+H]^+^ 515.1741 [M+Na]^+^ 537.1575 [2M+Na]^+^ 1051.3228	30 eV: 537.1757 (100) 50 eV: 173.0591 (100), 107.0476 (86), 201.0053 (69), 85.0275 (55), 229.0168 (49), 127.0540 (41), 145.0625 (36), 97.0267 (34)	Psilotinin-di-O-hexoside II	


BML-LCMS18-8.31-190.0627	190.0627/190.0631	0.20	C11H10O3	[M+H]^+^ 191.0703 [M+Na]^+^ 213.0528	30 eV: 173.0589 (100), 107.0491 (82) 50 eV: 107.0472 (100), 173.0592 (76), 145.0669 (18)	Psilotinin	


BML-LCMS18-11.20-594.1583	594.1583/594.1585	0.02	C27H30O15	[M+H]^+^ 595.1654 [M+Na]^+^ 617.1470	10 eV: no fragmentation 50 eV: 271.0583 (100)	Apigenin-6,8-di-C-glucoside (vicenin-2)	Markham, 1984; Authentic Standard


BML-LCMS18-13.99-1024.2462	1024.2462/1024.2485	2.21	C48H48O25	[M+H]^+^ 1025.2536 [M+Na]^+^ 1047.2336	10 eV: no fragmentation 50 eV: 539.0979 (100), 701.1499 (81), 269.1301(10)	Amentoflavone-tri-hexoside I	


BML-LCMS18-14.26-432.1067	432.1067/432.1056	2.44	C21H20O10	[M+H]^+^ 433.1130 [M+Na]^+^ 455.0945	10 eV: no fragmentation 50 eV: 271.0597 (100), 153.0175 (4)	Apigenin-7-O-glucoside (apigentrin, cosmosin)	Wallace and Markham, 1978; Authentic Standard


BML-LCMS18-14.50-578.1636	578.1636/5781636	0.00	C27H30O14	[M+H]^+^ 579.1712 [M+Na]^+^ 601.1520 [2M+Na]^+^ 1179.3113	10 eV: no fragmentation 50 eV: 271.0598 (100)	Apigenin-7-O-rhamnoglucoside (rhofolin)	Wallace and Markham, 1978


BML-LCMS18-14.82-1024.2480	1024.2480/1024.2485	0.03	C48H48O25	[M+H]^+^ 1025.2551 [M+Na]^+^ 1047.2352	10 eV: no fragmentation 50 eV: 539.0966 (100), 701.1482 (73), 863.1989 (14)	Amentoflavone-tri-hexoside II	


BML-LCMS18-15.78-862.1946	862.1946/862.1956	1.21	C42H38O20	[M+H]^+^ 863.2021 [M+Na]^+^ 885.1831 [M+K]^+^ 901.1516	10 eV: no fragmentation 50 eV: 539.0967 (100), 701.1482 (6)	Amentoflavone-di-hexoside I	


BML-LCMS18-16.60-862.1938	862.1938/862.1956	2.14	C42H38O20	[M+H]^+^ 863.2010 [M+Na]^+^ 885.1825	10 eV: no fragmentation 50 eV: 539.0963 (100), 701.1473 (34)	Amentoflavone-di-hexoside II	


BML-LCMS18-17.56-702.1582	702.1582/702.1585	0.38	C36H30O15	[M+H]^+^ 703.1642 [M+Na]^+^ 725.1487	10 eV: no fragmentation 50 eV: 421.0545 (100), 541.1130 (43), 311.0555 (17), 337.0343 (16), 271.0582 (10), 153.0175 (8), 137.0583 (7), 147.0434 (7), 297.0413 (6), 379.0452 (5)	Dihydroamentoflavone-hexoside I	


BML-LCMS18-18.12-700,1418	700,1418/700,1428	1.46	C36H28O15	[M+H]+ 701.1490 [M+Na]^+^ 723.1310	10 eV: no fragmentation 50 eV: 539.0976 (100), 403.0443 (12), 377.0654 (5)	Amentoflavone-hexoside I	


BML-LCMS18-18.28-702.1562	702.1562/702.1585	3.23	C36H30O15	[M+H]^+^ 703.1632 [M+Na]^+^ 725.1442	10 eV: no fragmentation 50 eV: 389.1015 (100), 541.1112 (65), 153.0958 (55), 415.0787 (20), 403.0439 (7)	Dihydroamentoflavone-hexoside II	


BML-LCMS18-19.16-700.1424	700.1424/700.1428	1.00	C36H28O15	[M+H]^+^ 701.1493 [M+Na]^+^ 723.1304	10 eV: no fragmentation 50 eV: 539.0965 (100), 403.0434 (19), 377.0658 (8), 153.017 (5)	Amentoflavone-hexoside II	


BML-LCMS18-19.96-554.0856	554.0856/554.0849	1.24	C30H18O11	[M+H]^+^ 555.0931	10 eV: no fragmentation 50 eV: 403.0641 (100), 153.0641 (94), 405.0951 (78), 377.0672 (70), 121.0297 (51), 347.0569 (40), 375.0844 (39), 335.0551 (36), 271.0600 (25), 283.0604 (24), 555.0880 (24)	Hydroxy-amentoflavone	Zhang Y.X. et al., 2011


BML-LCMS18-20.73-540.1053	540.1053/540.1057	0.64	C30H20O10	[M+H]^+^ 541.1125 [M+Na]^+^ 563.0948 [2M+Na]^+^ 1103.1994	10 eV: no fragmentation 50 eV: 311.0555 (100), 337.0350 (100), 283.0604 (94), 421.0558 (36), 312.0585 (19), 147.0433 (11), 335.055 (10), 253.0490 (8), 153.0189 (8),	2,3-Dihydro-amentoflavone	Zhang Y.X. et al., 2011 NMR (Table 2; F8)


BML-LCMS18-20.96-702.1573	702.1573/702.1585	1.67	C36H30O15	[M+H]^+^ 703.1641 [M+Na]^+^ 725.1468	10 eV: no fragmentation 50 eV: 541.1118 (100), 421.0543 (48), 393.0621 (10), 271.0578 (4)	Dihydroamentoflavone-hexoside III	


BML-LCMS18-21.15-538.0912	538.0912/538.0900	2.24	C30H18O10	[M+Na]^+^ 561.0785 [2M+H]^+^ 1077.1844 [2M+Na]^+^ 1099.1671	10 eV: no fragmentation 50 eV: 377.0657 (100), 403.0452 (98), 347.0551 (56), 335.0552 (55), 283.0600 (36), 153.0178 (34), 539.0964 (27), 121.0282 (25), 307.0601 (23), 387.0855 (21), 311.0549 (16)	Amentoflavone	Wallace and Markham, 1978; Wang et al., 2015; NMR (Table 2;F10); Authentic Standard


BML-LCMS18-21.41-540.1058	540.1058/540.1057	0.28	C30H20O10	[M+H]^+^ 541.1130 [M+Na]^+^ 563.0935	10 eV: no fragmentation 50 eV: 389.1016 (100), 153.0176 (86), 121.0281 (16), 270.0524 (10), 253.0485 (6), 377.0642 (6), 403.0457 (6), 347.0741 (5)	2″ 3″-Dihydro-amentoflavone	Zhang Y.X. et al., 2011


BML-LCMS18-22.03-583.4640	583.4640/583.4640	0.00	C30H18O10	[M+H]^+^ 539.0979	10 eV: no fragmentation 50 eV: 153.0165 (100), 387.0853 (93), 403.0447 (84), 521.0858 (76), 413.0647 (60), 377.0664 (56), 539.0966 (52), 270.0523 (49), 283.0592 (43), 347.0577 (42), 121.0324 (39), 389.0987 (34), 335.0987 (30), 311.0538 (30)	Robustaflavone	Zhang Y.X. et al., 2011; Wang et al., 2015; NMR (Table 2; F11)


BML-LCMS18-22.59-554.1201	554.1201/554.1213	2.16	C31H22O10	[M+H]^+^ 555.1270 [M+Na]^+^ 577.1097 [2M+Na]^+^ 1133.2517	10 eV: no fragmentation 50 eV: 167.0316 (100), 389.0992 (33), 257.0411 (22), 123.0427 (17), 270.0510 (11)	Dihydro-*O*-methyl-amentoflavone	Markham, 1984


BML-LCMS18-23.38-552.1052	552.1052/552.1056	0.81	C31H20O10	[M+H]^+^ 553.1127 [M+Na]^+^ 575.0941	10 eV: no fragmentation 50 eV: 89.0586 (100), 193.0483 (83), 149.0224 (76), 73.0284 (56), 237.0731 (56), 275.0447 (33), 285.0382 (22), 268.0692 (16), 254.0553 (13), 553.1074 (10), 286.0421 (7), 387.0759 (6)	*O*-Methyl-amentoflavone (tentative)	Markham, 1984; Wang et al., 2015


BML-LCMS18-23.80-542.1212	542.1212/542.1213	0.18	C30H22O10	[M+H]^+^ 543.1291 [M+Na]^+^ 565.1101 [2M+K]^+^ 1123.1935	10 eV: no fragmentation 50 eV: 153.0180 (100), 271.0592 (75), 391.1183 (26), 147.0427 (21), 297.0376 (10), 166.9972 (10), 179.0342 (10), 423.0729 (5)	Binaringenin	Feuereisen et al., 2017


BML-LCMS18-24.14-540.1060	540.1060/540.1057	0.65	C30H20O10	[M+H]^+^ 541.1136 [M+Na]^+^ 563.0941 [2M+Na]^+^ 1103.1995	10 eV: no fragmentation 50 eV: 153.1777 (100), 389.1015 (96), 257.0438 (53), 270.0514 (33), 285.0385 (18), 421.0533 (17),	Dihydrohinokiflavone (tentative)	


BML-LCMS18-24.50-538.0896	538.0896/538.0900	0.74	C30H18O10	[M+H]^+^ 539.0979 [M+Na]^+^ 561.0785 [2M+H]^+^ 1077.1844	10 eV: no fragmentation 50 eV: 539.0971 (100), 254.0571 (87), 270.0517 (76), 257.0441 (76), 242.0569 (56), 286.0460 (47), 387.0870 (34), 153.0165 (30)	Hinokiflavone	Zhang Y.X. et al., 2011; Wang et al., 2015 NMR (Table 2; F14)


### Metabolite Isolation and Analysis by Nuclear Magnetic Resonance Spectroscopy

Above-ground biomass from *P. nudum* was harvested and homogenized to a fine powder in the presence of liquid nitrogen. A 300 mg aliquot of the homogenate was extracted with 10 ml of 80% aqueous methanol by vigorous mixing for 10 min (Vortex Mixer, VWR Scientific, South Plainfield, NY, United States; operated at highest speed setting) and subsequent sonication in an ultrasonic bath for 20 min. Following centrifugation of this mixture for 10 min at 13,000 rpm, the supernatant was recovered and filtered through a 0.22 μm polypropylene filter. The extract was stored at –20°C until further processing. Aliquots (100 μl each) of the filtered extracts were injected onto a C18 reversed phase and absorbance at 280 and 360 nm was monitored (1100 Series HPLC system; Agilent Technologies, Santa Clara, CA, United States). The mobile phase consisted of two solvents (A: 0.2% (v/v) acetic acid in water; B: 0.2% (v/v) acetic acid in methanol) and the separation of metabolites was achieved using the following gradient: 2% B at start, with a series of linear gradients to 35% B at 10 min, 60% B at 21 min, 90% B at 40 min, and 98% B at 50 min. The flow rate was set to 1.3 ml/min. Trial runs indicated when metabolites of interest eluted and fractions were collected accordingly. The eluents of several runs were accumulated and each of these fractions evaporated to dryness in a rotary evaporator. Each residue was dissolved in a deuterated solvent and NMR spectra were acquired with the settings listed in Supplementary Table S4. Spectral records for bioflavonoids and arylpyrones were generated based on information extracted from the literature (listing in Supplementary Table S5) and integrated into the Spektraris database (Cuthbertson et al., 2013; Fischedick et al., 2015). The combined spectral data from HPLC–QTOF–MS and NMR were then used to search for matches in the Spektraris online resource (Table 2).

**Table 2:** Peak annotation based on Spektraris searches with combined HPLC-QTOF-MS and NMR spectroscopy data.

Accurate Mass – Time Tag	Molecular Formula	NMR Solvent	NMR Signals (chemical shift, with integral, signal multiplicity, coupling constant and position in parentheses)	Spektraris Search Score (out of 100)	Annotation	References; Further Evidence
BML-LCMS18-4.66-368.1101	C17H20O9	D_2_O	7.21 (1H, d, 8 Hz, H-5’), 7.13-7.11 (1H, m, H-4), 6.94 (1H, d, 4 Hz, H-2’), 6.87 (1H, dd 8 and 7 Hz, H-6’), 6.07 (1H, dd, 12 and 4 Hz, H-3), 5.42 (1H, dd, 8 and 4 Hz, H-6), 5.0 (1H, m, H-1’) 3.89 (1H, dd, 8 and 4 Hz, H-6), 3.72 (1H, dd, 16 and 4 Hz, H-6), 3.63 (4H, m), 2.64 (2H, m, H-5a,b)	96	3’-Hydroxypsilotin	Balza et al., 1985
BML-LCMS18-5.20-352.1168	C17H20O8	CD_3_OD	7.4 (1H, d, 4 Hz, H-2), 7.11 (1H, m, H-4), 6.08 (1H, ddd, 16, 8 and 4 Hz, H-3), 5.48 (1H, dd, 12 and 4 Hz, H-6), 3.89 (1H, dd, 12 and 4 Hz, H-6A’), 3.72 (1H, dd, 12 and 4 Hz, H-6B’), 3.5 (4H, m), 2.65 (2H, m, H-5a,b)	90	Psilotin	McInnes et al., 1965
BML-LCMS18-20.73-540.1053	C30H20O10	DMSO-d6	7.58 (2H, d, 12 Hz, H-2’″ and H-6’″), 7.45 (2H, m, H-2’ and H-6’) 7.04 (1H, d, 8 Hz, H-5’), 6.79 (2H, d, 8 Hz, H-3’″ and H-5’″), 6.59 (1H, s, H-3″), 6.33 (1H, s, H-6″), 5.85 (2H, m, H-6 and H-8), 5.47 (1H, d, 4 Hz, H-2), 3.15(1H, m, H-3a), 2.75(1H, m, H-3b)	82	2,3-Dihydro-amentoflavone	Zhang Y.X. et al., 2011
BML-LCMS18-21.15-538.0912	C30H18O10	DMSO-d6	8.01 (1H, dd, 8 and 4 Hz, H-6’), 7.94 (1H, d, 4 Hz, H-2’), 7.58 (2H, d, 8 Hz, H-2’″ and H-6’″), 7.13 (1H, d, 8 Hz, H-5’), 7.01 (1H, s,H-3), 6.8(1H, s, H-3″), 6.7 (2H, d, 8 Hz, H-3’″ and H-5’″), 6.63 (1H, d, 8 Hz, H-8), 6.35 (1H, s, H-6″), 6.21 (1H, d, 4 Hz, H-6)	99	Amentoflavone (3’,8″-biapigenin)	Geiger et al., 1993
BML-LCMS18-22.03-583.4640	C30H18O10	DMSO-d6	7.9 (3H, m, H-6’, H-2’″ and H-6’″), 7.2 (1H, d,4Hz, H-2’), 6.95 (1H, d, 4Hz, H-5’), 6.91 (1H, d, 16 Hz, H-3’″ and H-5’″), 6.82(1H, s, H-3). 6.73 (1H, s, H-3″), 6.71 (1H, s, H-8″), 6.43 (1H, d, 4 Hz, H-8), 6.14 (1H, d,4Hz, H-6)	87	Robustaflavone (3’,6″-biapigenin)	Geiger et al., 1993
BML-LCMS18-24.50-538.0896	C30H18O10	DMSO-d6	7.9 (3H, m, H-2’, H-2’″ and H-6’″), 7.18 (2H, d, 4 Hz, H-3’ and H-6’), 6.92 (2H, d, 4Hz, H-3’″ and H-5’″) 6.88 (3H, m, H-3, H-5’ and H-3″), 6.75 (1H, s, H-3’), 6.72 (1H, s, H-8″), 6.41 (1H, d, 4 Hz, H-8), 6.13 (1H, d, 4Hz, H-6)	81	Hinokiflavone (4’,6″-O-biapigenin)	Geiger et al., 1993


### Metabolite Imaging by Matrix-Assisted Laser Desorption/Ionization – Mass Spectrometry

*Psilotum nudum* above-ground rhizomes were cross-sectioned into 2 cm segments, embedded in 3% (w/v) agarose, and stored at –80°C until further processing. On the day of the metabolite imaging analysis, the chamber of a CM 1950 Cryostat (Leica Biosystems, Buffalo Grove, IL, United States) was set to –20°C, embedded samples were sectioned to 30 μm thickness and sections immediately transferred to an imaging target plate (Waters Corp., Milford, MA, United States). The ionization matrices tested for their suitability with *P. nudum* metabolites were 2,5-dihydroxybenzoic acid (DBA), α-cyano-4-hydroxycinnamic acid (CHCA), sinapic acid and vanillic acid (each at 40 mg/ml (w/v) in methanol/water (1:1; v/v)). Matrices were applied with a sample preparation system (TM-Sprayer of HTX Technologies, Chapel Hill, NC, United States) connected to an 1100 Series HPLC Binary Pump (Agilent Technologies, Santa Clara, CA, United States). The settings were: flow rate at 0.05 ml/min; nozzle temperature at 80°C; spraying velocity at 1,250 mm/min; 12 passes; and track spacing of 1 mm. The final amount of matrix deposited per linear distance was 0.19 mg mm^-2^. Besides matrix-covered samples, the following chemicals were also spotted onto the imaging target plates: red phosphorus for instrument calibration (10 mg/ml in acetone), leucine-enkephalin to generate a lock mass [10 mg/ml mixed with 3.4 mg/ml CHCA in methanol/water (1:1; v/v)], and authentic standards [1 mg/ml of amentoflavone, psilotin and 3’-hydroxypsilotin mixed with 5 mg/ml DHB in methanol/water (1:1; v/v)]. Metabolite imaging was performed by Matrix-Assisted Laser Desorption/Ionization Mass Spectrometry (MALDI–MS) on a Synapt G2-S instrument equipped with an ion mobility drift tube and operated with MassLynx software version 4.1 (Waters, Milford, MA, United States). The imaging target plate was introduced into the sample chamber and the laser operated with the following settings: 1,000 Hz firing rate; laser energy of 40 (arbitrary units); and a step size of 25 μm. Lock mass correction was repeated every 600 s for a duration of 5 s. Other settings: helium gas flow at 90 ml min^-1^; trap wave velocity at 311 m s^-1^; trap wave height at 4 V; ion mobility wave velocity at 650 m s^-1^; ion mobility wave height at 40 V; transfer ware velocity at 191 m s^-1^; transfer wave height of 0.1 V; and ion mobility wave delay of 450 μs. The highest signal intensity for the analytes of interest (and thus most desirable signal-to-noise ratio) was achieved in positive polarity for psilotin and 3’-hydroxypsilotin, whereas for amentoflavone negative polarity was preferable. MS/MS experiments were performed by selecting a precursor ion and a collision energy of 30 eV in the transfer cell. MALDI–MS data were processed using the High Definition Imaging software version 1.2 (Waters, Milford, MA, United States) with lock mass correction. Metabolite identification was achieved by comparing accurate mass, MS/MS fragmentation patterns, and ion mobility drift time with those of authentic standards. Signals for isomers of amentoflavone, for example robustaflavone and hinokiflavone, were detectable with authentic standards but not in tissue samples, where their concentrations were too low for MS-based imaging.

## Results

### Strategy for Multi-Platform Analysis of Metabolites in *P. nudum* Organs

**FIGURE 1:**
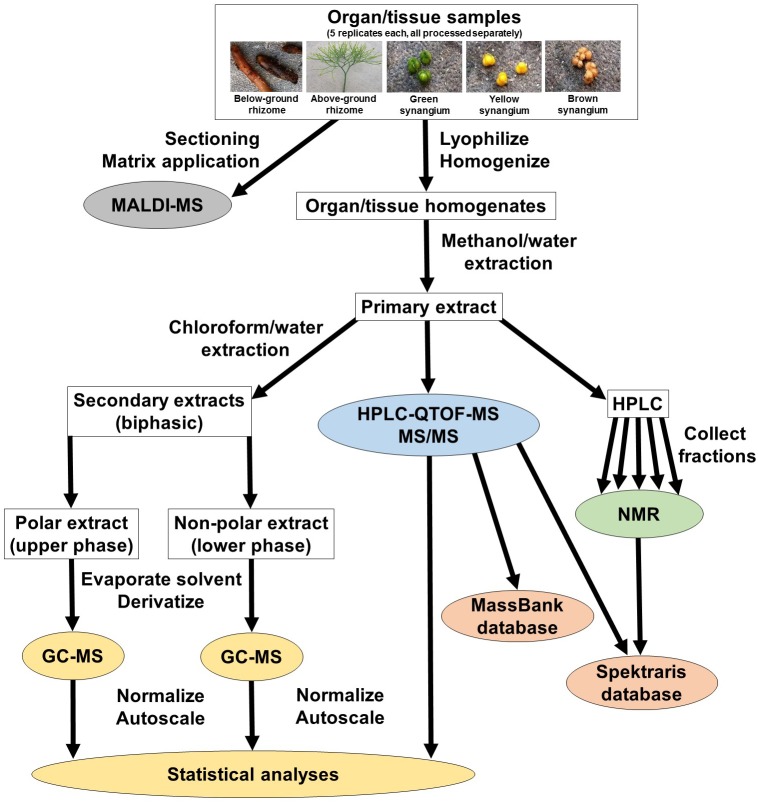
Experimental design for the current study. For details and abbreviations see text.

In an attempt to capture chemically diverse metabolites, we used a strategy that accessed five *P. nudum* organs/tissues (below-ground rhizome, above-ground rhizome, and synangia harvested at different developmental stages [green (young), yellow (maturing) and brown (mature)], and generated hydrophilic (methanol/water) and hydrophobic (chloroform/methanol) extracts (Figure 1). These two types of extracts were processed separately for GC–MS analysis (Supplementary Figure S1). Methanol/water extracts were also subjected to HPLC–QTOF–MS analysis in positive ionization mode only (preliminary experiments indicated that chromatographic runs in negative polarity did not add significant spectral information) (Supplementary Figure S1). These data sets were normalized, autoscaled, and then combined for multivariate statistical analyses (Figure 1). Fractions representing selected metabolites of interest were collected from chromatographic separations of extracts and further characterized by ^1^H-NMR. The different metabolomics platforms (GC–MS and HPLC–QTOF–MS) were chosen because they provide complementary information about different metabolites classes (details presented in the upcoming paragraphs). Cryosections of *P. nudum* above-ground rhizomes were sprayed with a chemical matrix and the cell type-level localization of the most abundant metabolites determined by MALDI–QTOF–MS (Figure 1).

**FIGURE 2:**
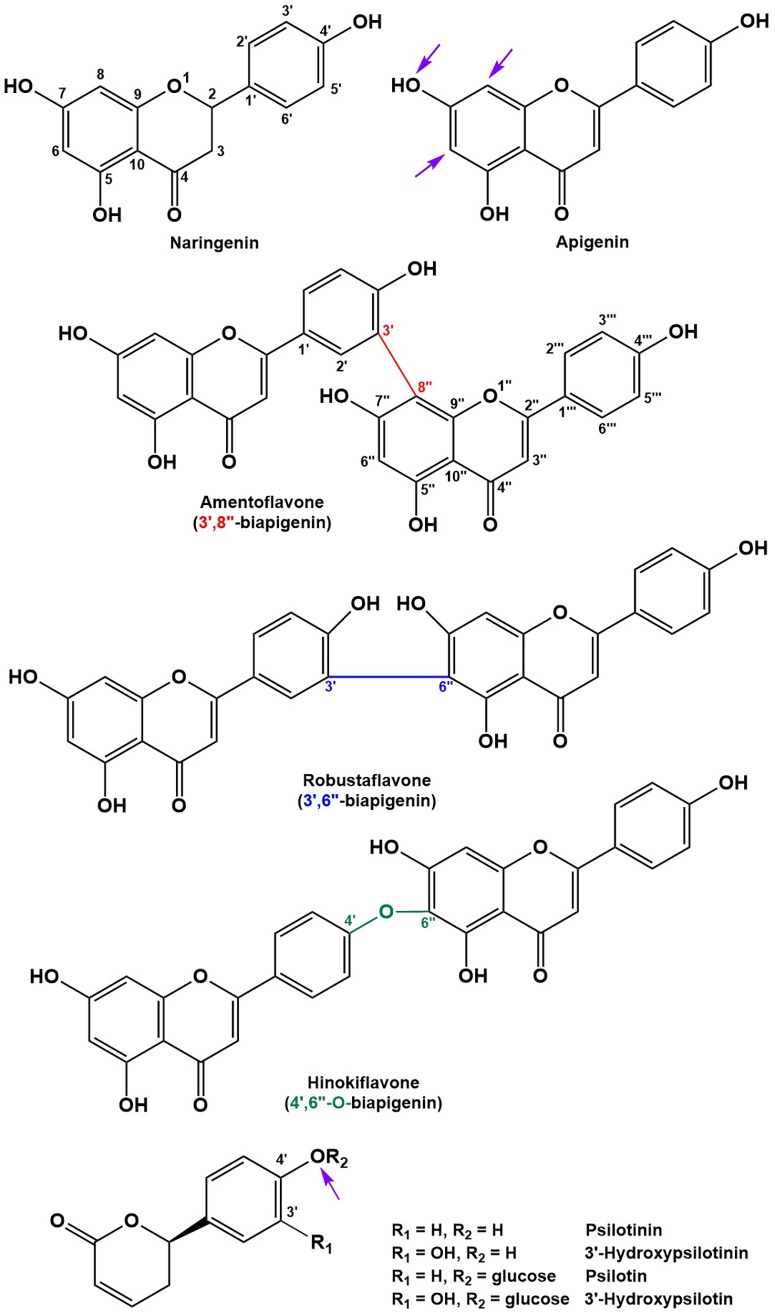
Structures of the main flavonoid, biflavonoid and arylpyrone agylcones of *P. nudum* (positions for common glycosylation sites are indicated with purple arrows).

Peak annotation for GC–MS data was achieved based on comparisons of retention times and mass spectral characteristics with those of authentic standards, which led to the high-confidence identification of 83 metabolites in our extracts. MS and MS/MS data from HPLC–QTOF–MS runs (acquired in positive polarity mode) were searched against comprehensive online databases (MassBank^2^, Metlin^3^, and National Institute of Standards and Technology^4^). However, these searches were mostly unsuccessful due to a lack of relevant reference spectra in these databases, and we therefore decided to expand the Spektraris online resource^5^ with spectral records acquired as part of this study or extracted from information in the literature. Accurate mass, inferred molecular formula, NMR spectral data, and bibliographic information for 328 metabolites (arylpyrone and biflavonoid aglycones and corresponding glycosides) were integrated into Spektraris-NMR, which now contains spectral records for approximately 21,500 metabolites (status: February, 2019). The combination of accurate mass and retention time data (AMT-tags) acquired by HPLC–QTOF–MS were then searched against Spektraris records (for details of this approach see Cuthbertson et al., 2013), which provided tentative identifications for 27 metabolite peaks (4 annotations with high confidence because of available authentic standards) (Table 1). By also including ^1^H-NMR data in Spektraris searches (Fischedick et al., 2015), eight metabolites could be identified with high confidence (Table 2). Relevant structures of arylpyrones and bioflavonoids are shown in Figure 2 and the annotation process for selected peaks is outlined in more detail in the following paragraph.

Arylpyrone glycosides with a psilotinin aglycone eluted early (4.6–6.9 min), followed by flavonoid glycosides (11.2–14.6 min), biflavonoid glycosides (14.0–19.3 min), and then biflavonoid aglycones (19.9–24.7 min). In addition to the quasi-molecular ion ([M+H]^+^), adducts ([M+Na]^+^ and [M+K]^+^) and dimers ([2M+Na]^+^ and [2M+K]^+^) were detected consistently for almost all analytes (Table 1). An authentic standard of psilotin (R_t_ 5.20 min; m/z 353.1250 ([M+H]^+^); C_17_H_20_O_8_) (McInnes et al., 1965) allowed us to investigate the typical fragmentation patterns of this class of arylpyrone glycosides. At low fragmentation energy (10 eV), the loss of glucose generated a fragment representing the psilotinin aglycone (m/z 191.0699; [M–Glc]^+^; C_11_H_10_O_3_), a second fragment with additional loss of water (m/z 173.0598; [M–Glc–H_2_O]^+^; C_11_H_10_O_3_), a third fragment at m/z 123.0438 (C_7_H_7_O_2_), and a fourth fragment with m/z 107.0487 (C_7_H_7_O). The absorption spectrum of the peak in chromatograms and the authentic psilotin standard was essentially identical (Supplementary Figure S2). Furthermore, following purification of the peak by HPLC, the NMR spectrum of the isolated metabolite matched literature reports (McInnes et al., 1965) (Table 2). The signature fragments generated from the psilotin HPLC–QTOF–MS peak (corresponding to C_11_H_10_O_3_ and C_7_H_7_O) were also detected in MS/MS spectra of four additional peaks (plus further common fragments at 50 eV). The molecular ions of two of these peaks indicated the potential presence of two hexose moieties (R_t_ 5.12 and 6.83 min; m/z 515.1751 ([M+H]^+^); C_23_H_30_O_13_), which was corroborated by fragmentation patterns ([M – 2 × Glc]^+^ and [M – 2 × Glc – H_2_O]^+^). The third peak of this series had a molecular ion consistent with 3’-hydroxypsilotin (R_t_ 4.66 min; m/z 369.1198 ([M+H]^+^); C_17_H_20_O_9_) (Balza et al., 1985), an annotation that was confirmed based on the NMR spectrum of the isolated metabolite (Tables 1, 2). The fourth peak appeared to contain the same aglycone but with two attached hexose moieties and was therefore tentatively annotated as 3’-hydroxypsilotinin-di-O-hexoside (R_t_ 4.76 min; m/z 553.1530 ([M+Na]^+^); C_23_H_30_O_14_).

Based on the characteristics of the peak corresponding to apigenin 7-O-glucoside, for which an authentic standard was available (R_t_ 14.26 min; m/z 433.1133 ([M+H]^+^); C_21_H_20_O_10_) (Wallace and Markham, 1978), the fragment indicative of an apigenin aglycone was m/z 271.0597 ([M–Glc]^+^; C_15_H_10_O_5_), with m/z 153.0175 (C_7_H_5_O_4_) representing a second prominent fragment obtained from this flavone aglycone (Kachlicki et al., 2016). Two additional peaks had comparable fragmentation patterns, one of which showed a quasi-molecular ion corresponding to apigenin-7-O-rhamnoglucoside (R_t_ 14.50 min; m/z 579.1712 ([M+H]^+^); C_27_H_30_O_14_), a metabolite that had previously been reported to occur in *P. nudum* (Wallace and Markham, 1978). The mass spectrum of the second of these peaks was indicative of a metabolite with two hexose moieties (R_t_ 11.20 min; m/z 595.1654 ([M+H]^+^); C_27_H_30_O_15_) and therefore likely corresponds to an apigenin di-hexoside. Interestingly, apigenin-6,8-di-C-glucoside (vicenin-2) was described before as an abundant constituent of *P. nudum* extracts (Markham,1984), which was used as a tentative annotation for the peak of interest (Table 1).

All biflavonoid glycosides thus far characterized from *P. nudum* extracts contained an amentoflavone (3′,8″-biapigenin) aglycone with likely O-linked hexose moieties (Wallace and Markham, 1978; Markham, 1984). The amentoflavone authentic standard (C_30_H_18_O_10_) eluted at 21.15 min (Table 1). Six additional peaks with the characteristic m/z 539.0963 fragment (corresponding to this aglycone) plus common MS/MS fragments of the aglycone (Zhang Y.X. et al., 2011; Feuereisen et al., 2017) were detected in our extracts. Based on their quasi-molecular ion, these peaks were tentatively annotated as amentoflavone-hexosides (R_t_ 18.12 and 19.16 min; m/z 701.1490 ([M+H]^+^); C_36_H_28_O_15_), amentoflavone-di-hexosides (R_t_ 15.78 and 16.60 min; m/z 863.2021 ([M+H]^+^); C_42_H_38_O_20_) or amentoflavone-tri-hexosides (R_t_ 13.99 and 14.82 min; m/z 1025.2551 ([M+H]^+^); C_48_H_48_O_25_). Three peaks in the same retention time region (R_t_ 17.56, 18.28, and 20.96 min) had a quasi-molecular ion (m/z 703.1642 ([M+H]^+^); C_36_H_30_O_15_) and characteristic aglycone fragment (m/z 541.0545; C_30_H_20_O_10_) indicative of two additional mass units compared to amentoflavone-hexosides. The aglycone in these cases is dihydroamentoflavone (C–C-linked dimer of apigenin and naringenin), and the peaks were therefore annotated as dihydroamentoflavone-hexosides (Table 1). The absorption spectrum of the amentoflavone standard in the ultraviolet and visible range was identical to that of the corresponding peak in our HPLC runs (Supplementary Figure S2). Two arylpyrone aglycones, 3′-hydroxypsilotinin (R_t_ 5.52; m/z 207.0652 ([M+H]^+^); C_11_H_10_O_4_) and psilotinin (R_t_ 8.31; m/z 191.0703 (M+H]^+^); C_11_H_10_O_3_), were tentatively identified based on chromatographic properties and similarity of MS/MS fragmentation patterns to those of their glycosides (Supplementary Figure S3).

To enable the differentiation of biflavonoid aglycones, four fractions collected by HPLC were subjected to ^1^H-NMR spectroscopy. Taking into account the previously published elution order of biflavonoids on reversed-phase HPLC materials (Zhang Y.X. et al., 2011), MS and MS/MS data, and by combining this information with MS and NMR data searches against the Spektraris database, four peaks could be identified with high confidence (20.73 min, 2,3-dihydroamentoflavone; 21.15 min, amentoflavone; 22.03 min, robustaflavone; and 24.50 min, hinokiflavone (Figure 2 and Tables 1, 2). The peak at 19.96 min (m/z 555.0931 ([M+H]^+^); C_30_H_18_O_11_) was tentatively identified as hydroxyamentoflavone based on its earlier elution (compared to amentoflavone) and MS/MS fragmentation patterns (Zhang Y.X. et al., 2011). Analogous comparisons allowed the tentative identification of dihydro-*O*-methyl-amentoflavone (Rt 22.59 min; m/z 555.1270 ([M+H]^+^); C_31_H_22_O_10_), *O*-methyl-amentoflavone (Rt 23.38 min; m/z 553.1127 ([M+H]^+^); C_31_H_20_O_10_), binaringenin (Rt 23.80 min; m/z 543.1291 ([M+H]^+^); C_30_H_22_O_10_), and dihydrohinokiflavone (Rt 24.14 min; m/z 541.1136 ([M+H]^+^); C_30_H_20_O_10_) (Markham, 1984; Zhang Y.X. et al., 2011; Wang et al., 2015; Feuereisen et al., 2017) (Table 1).

A third class of metabolites with high abundance in HPLC–QTOF–MS runs had the chromatographic and mass spectral properties of highly functionalized triterpenoids (steroids) (Supplementary Table S2). While the typical membrane sterols of *P. nudum* have been reported before (Akihisa et al., 1992), the more functionalized steroids detected here have not been mentioned in previous studies. A more detailed characterization of these underexplored specialized metabolites will be the subject of future endeavors to further evaluate chemicals diversity in the fern lineage.

### Principal Component Analysis Differentiates Metabolomics Data Sets From Different *P. nudum* Organs

**FIGURE 3:**
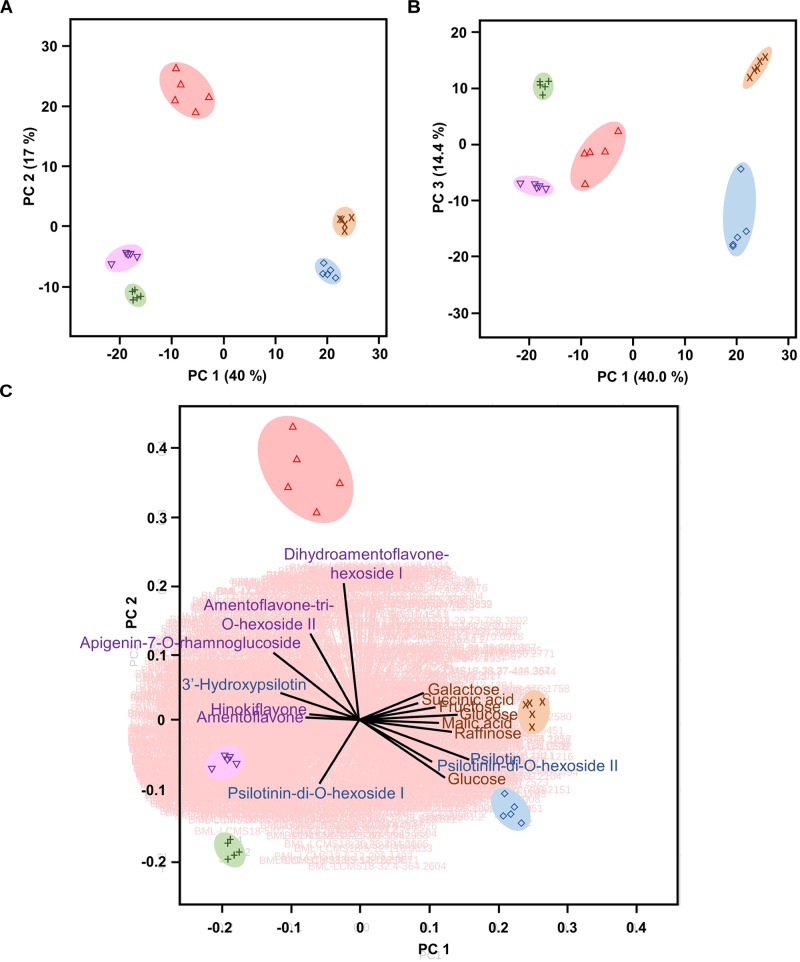
*Psilotum nudum* organs have a unique metabolic fingerprint, based on PCA of combined GC-MS and HPLC-QTOF-MS data. **(A)** PCA plot for PC1 and PC2. **(B)** PCA plot for PC1 and PC3. **(C)** Bi-plot with selected component loadings (metabolites) highlighted (the vectors of the remaining AMT tags are shown with red lines in the background). The full component loadings are provided in Supplementary Table S3. Symbols: rhizome (below-ground), purple cross; rhizome (above-ground), blue diamond; green synangium, green plus sign; yellow synangium, pink inverted triangle; brown synangium, red triangle.

Multivariate statistical analyses, such as PCA, aid with reducing the complexity of extensive data sets into a smaller number of Principal Components (PCs). When this approach was brought to bear on our combined GC–MS and HPLC–QTOF–MS data sets, the first three PCs accounted for roughly 71% of the varied influences of the original characteristics (metabolite patterns across all sample types), and indicated a clear separation of the five sample types (*P. nudum* below-ground rhizome, above-ground rhizome, and synangia harvested at three different developmental stages), with a tight clustering of biological replicates (Figure 3A,B). Below-ground rhizome samples were characterized by positive scores in PC1 and PC3, with neutral values in PC2. Above-ground rhizome samples also had positive scores in PC1 but negative scores in PC2 and PC3 (Figure 3A,B). All samples from synangia had negative scores in PC1, but were differentiated by a combination of negative PC2/positive PC3 scores (green synangia), negative PC2/PC3 scores (yellow synangia) or positive PC2/neutral PC3 scores (brown synangia) (Figure 3A,B).

Component loadings were then evaluated for characteristics that contributed to the differences among sample clusters in PCA and visualized in a biplot (Figure 3C and Supplementary Table S3). The scores for below-ground rhizome samples (positive PC1 and PC2) corresponded to the vectors for carbohydrate monomers and dimers (e.g., glucose, fructose, galactose, and raffinose) and small organic acids (malic acid, citric acid, and succinic acid). Above-ground rhizome samples occupied a biplot position (positive PC1, negative PC2) closer to the direction of vectors for some arylpyrone glycosides (psilotin and psilotinin-di-O-hexoside II) and sucrose (which had high concentrations in both above- and below-ground rhizomes) (Figure 3C). The unique position of brown synangia (negative PC1/positive PC2) correlated with the vector for biflavonoid glycosides (e.g., dihydroamentoflavone hexoside I and amentoflavone-tri-O-hexoside II). Scores for green and yellow synangia were similar (negative scores in both PC1 and PC2) and correlated with the direction of vectors for arylpyrone glycosides (e.g., psilotinin-di-O-hexoside I) and biflavonoid aglycones (e.g., amentoflavone and hinokiflavone) (Figure 3C).

### Organ-Specific Accumulation of Metabolites

**FIGURE 4:**
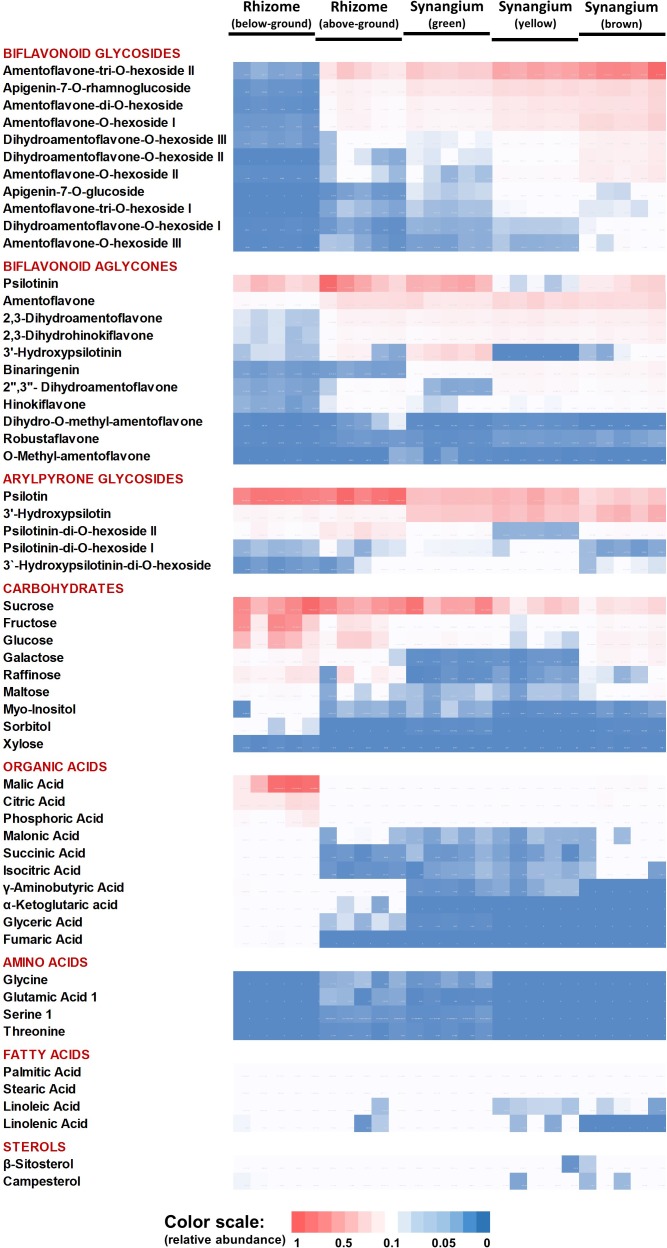
Heat map visualizing the organ-specificity of metabolite accumulation in *P. nudum*. A color code (red-white-blue) is used to indicate the relative abundance of metabolites based on normalized mass spectral intensities.

The PCA component loadings indicated that specific metabolite classes might explain the separation of sample types. We therefore generated a heatmap of metabolite accumulation patterns across *P. nudum* organs (Figure 4). The relative quantities of five biflavonoid glycosides, based on normalized peak areas, were quite high in brown synangia, followed by yellow and green synangia. These metabolites were also of fairly high abundance in samples of above-ground rhizomes, but extremely low in below-ground rhizomes (Figure 4). The quantities of six additional biflavonoid glycosides were considerably lower in all samples. Amentoflavone was the by far most abundant biflavonoid aglycone, with very high amounts present in yellow and brown synangia, relatively high quantities in above-ground rhizomes and green synangia, and fairly low levels in below-ground rhizomes (Figure 4). Similar patterns were observed for five additional biflavonoid aglycones (three dihydrobiapigenin isomers, binaringenin and hinokiflavone), albeit at much lower abundance compared to amentoflavone. Among arylpyrone glycosides, psilotin was most abundant in rhizomes and above-ground rhizomes, but was also accumulated to appreciable amounts in synangia (Figure 4). 3′-Hydroxypsilotin was primarily found in synangia, with an abundance comparable to that of psilotin. Three other arylpyrone glycosides were of relatively low abundance in all samples.

Sucrose was equally abundant in rhizomes, above-ground rhizomes and green synangia (Figure 4). Glucose, fructose and other small molecule carbohydrates were most abundant in rhizomes, with significantly lower amounts being present in all other samples. The highest levels of small organic acids were also found in rhizomes. While malic and citric acid were fairly abundant in all samples, other organic acids (e.g., α-ketoglutaric acid, glyceric acid and fumaric acid) were detected at considerably lower levels in rhizomes and yet lower levels in all other samples (Figure 4).

### MALDI–MS Imaging Indicates Preferential Accumulation of Amentoflavone and Arylpyrone Glycosides in Stem Epidermis and Outer Cortex

**FIGURE 5:**
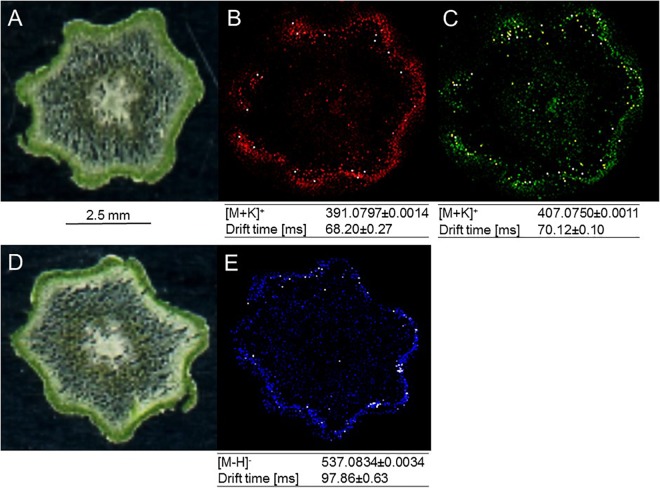
MALDI-MS imaging of specialized metabolites in cross sections of *P. nudum* above-ground rhizomes (stems). **(A)** Image of specimen for arylpyrone glycoside analysis; **(B)** [M+K]^+^ signal for psilotin (colored in red); **(C)** [M+K]^+^ signal for 3′-hydroxypsilotin (colored in green); **(D)** image of specimen for biflavonoid analysis; **(E)** [M–H]^-^ signal for amentoflavone (colored in blue). Note the accumulation of metabolites of interest in the chlorenchyma (dark green tissue in specimen images).

Building on recent successes with MS-based imaging of sesquiterpene alkaloids and triterpenoids (Lange et al., 2017), MALDI–MS was employed for localizing metabolites of interest in the current study. Two arylpyrone glycosides, psilotin and 3′-hydroxypsilotin, and a biflavonoid aglycone, amentoflavone, were selected for because they were highly abundant in tissue samples (MS-based imaging is much less sensitive compared to tissue extraction followed by HPLC–QTOF–MS) and were available as authentic standards in sufficient quantities for methods development. Based on the results of preliminary experiments, 30 μm cryosections of above-ground rhizomes served as biological material, 2,5-dihydroxybenzoic acid was chosen as matrix substance to aid with ionization of metabolites desorbed from tissue sections, and leucine-enkephalin was selected to provide an external lock mass. The ionization of psilotin and 3′-hydroxypsilotin was most effective in positive ionization mode, where potassium adducts (m/z 391.0797 and 407.0750, respectively) were readily detectable with unique drift times in the ion mobility cell. Mass spectrometric signals for psilotin, 3′-hydroxypsilotin and amentoflavone were highest in the epidermal and outer cortex layers, which collectively form the chlorenchyma (Figure 5A–D). The 3′-hydroxypsilotin signal was also apparent, albeit at significantly lower abundance, in the protostele. Amentoflavone ionized particularly well in negative mode, with the quasi-molecular ion being more abundant than adducts (m/z 537.0827) and traveling through the ion mobility cell with a unique drift time (Figure 5B,C,E). Based on MALDI–MS experiments performed with above-ground rhizome extracts, the normalized peak area for amentoflavone was 5-fold higher than that of hinokiflavone and 47-fold higher than that of robustaflavone, and the abundance of the latter two metabolites was too low for localization studies.

## Discussion

### Expanding the Coverage of Spectral Databases to Incorporate Information on Chemical Diversity in the Fern Lineage

Biflavonoids have long been known to accumulate prominently across the bryophytes, pteridophytes and gymnosperms, with only sporadic occurrence in the angiosperms (Geiger and Quinn, 1988; Iwashina, 2000). When we began processing the data presented as part of the current study with *P. nudum*, we noticed a surprising paucity of spectral data relating to biflavonoids in publicly available MS and NMR databases. We therefore embarked on a literature search to gather phytochemical and spectral data for this important class of metabolites, which was then used to generate 328 new spectral records for the Spektraris online resource (Cuthbertson et al., 2013; Fischedick et al., 2015). Additionally, electronic files representing the MS/MS data acquired with biflavonoids were submitted to MassBank, a widely used online mass spectral repository (Horai et al., 2010).

The orthogonal data sets acquired in this study (retention time on GC or HPLC, quasi-molecular ion (and inferred molecular formula), MS/MS data, and NMR spectra), combined with the use of authentic standards, aided substantially in peak annotation. The inclusion of NMR data was particularly impactful for the annotation of peaks for the biflavonoids (amentoflavone, robustaflavone, and hinokiflavone) that consist of two fused apigenin molecules (differing only in the coupling position). Using our integrative approach, a total of 83 GC-MS and 8 HPLC–QTOF–MS peaks were identified with very high confidence. An additional 23 HPLC–QTOF–MS peaks were tentatively identified (for example, amentoflavone-tri-O-hexoside I, where uncertainty pertains only to the position and exact nature of the hexose moiety) (Table 1). While we were able to determine the structures of some of the more abundant aglycones, the identification of biflavonoid glycosides, which occur as larger families of closely related structures, has proven much more difficult. Our data sets also contained a very large number of peaks that could not be identified. Some of these, based on peak area counts, appeared to be fairly abundant. These results indicate that significant efforts will be needed to generate a more comprehensive account of chemical diversity in *P. nudum* and, more broadly, in the fern lineage.

### Below-Ground Rhizome of *P. nudum* Contains High Levels of Soluble Sugars and Organic Acids, Possibly Indicating Differential Nutrient Allocation

The above- and below-ground portions of the *P. nudum* rhizome are part of the same organ and it is thus notable that, in our study, significantly higher amounts of soluble sugars (in order of abundance: fructose, glucose, raffinose and galactose) and organic acids (in order of abundance: malic acid, citric acid and phosphoric acid) were present in the below-ground part of the rhizome. The abundance of soluble sugars might be interpreted as evidence for a storage function for *P. nudum* rhizomes but, to the best of our knowledge, the corresponding storage sugar polymers have not been analyzed in this species. The chemical properties of rhizome starches have been reported for other ferns (Zhang S. et al., 2011; Yu et al., 2015) and this work indicates indirectly (based on the high abundance of sugar precursors) that storage function is a possibility. It is also conceivable that relatively high levels of soluble sugars and organic acids are a reflection of active metabolism to support horizontal rhizome growth in *P. nudum*. However, while information is available regarding the correlation of fern development and some classes of metabolites (White and Turner, 1995; Abul et al., 2010), we were not able to find literature on soluble sugar quantities in fern rhizomes. Further research is clearly necessary to begin to appreciate the tissue specialization within fern rhizomes.

### Rhizomes Accumulate Particularly High Amounts of Psilotin, an Arylpyrone Glycoside With Demonstrated Biological Activities

Our data indicated that psilotin and psilotinin, both arylpyrones unique to the Psilotaceae, were most abundant in the rhizome (both below- and above-ground), while being only half or one-third as abundant in samples from synangia. The biflavonoid amentoflavone was also highly abundant in the above-ground part of the rhizome but occurred at fairly low quantities in the below-ground parts (Figure 4). This begs the question if psilotin and its aglycone psilotinin might play a particular role in the below-ground rhizome, where arylpyrones are major constituents. Interestingly, it was demonstrated more than 40 years ago that psilotin acts as a germination inhibitor for turnip, onion and lettuce seeds (Siegel, 1976). It is, therefore, conceivable that psilotin (and possibly its aglycone as well) plays a defensive or allelochemical role in and around the below-ground rhizome. Psilotin was also shown to have antifeedant activities against the European corn borer (*Ostrinia nubilalis*) at concentrations below those present in *P. nudum* (Arnason et al., 1986). However, in the absence of more complete data on the bioactivities of arylpyrones, this interpretation is highly speculative. It is also unknown how psilotin might be secreted into the rhizosphere to exert allelochemical activities. The fact that the inhibitory effects of psilotin on germination can be reversed by the addition of GA_3_ (Siegel, 1976), a gibberellin hormone, can be interpreted as evidence for a possible role of this arylpyrone in growth regulation, but the mechanism and target(s) of such an activity have not yet been explored. In the above-ground rhizome, psilotin and amentoflavone (the latter also exerting high bioactivity; Yu et al., 2017) may act collectively as defense metabolites. Currently, information about such activities has been inferred from *in vitro* assays only and it would thus be informative to also assess potential defensive functions of arylpyrones and bioflavonoids in *in vivo* investigations.

### Occurrence of Biflavonoids and Arylpyrones in Chlorenchyma Is Consistent With Function as Sunscreen Pigments

Based on our MALDI–MS imaging data, psilotin and amentoflavone are accumulated preferentially in the photosynthetically active tissues of above-ground rhizomes (above-ground rhizomes) (Figure 5). Considering the absorption characteristics of these metabolites (Supplementary Figure S2), a protective function against excess photosynthetically active radiation and certain wavelengths (e.g., high energy ultraviolet-B) radiation would be a reasonable hypothesis for their tissue-level localization (Yamaguchi et al., 2009; Waterman et al., 2017). Our localization data sets for amentoflavone (chlorenchyma) are also consistent with the literature for other plants. For example, amentoflavone was accumulated preferentially in the leaf epidermis in *Agathis robusta* (Gadek et al., 1984) and *Ginkgo biloba* (Beck and Stengel, 2016). An interesting, as yet unanswered, question pertains to the functional role of the differential subcellular localization one would predict for the metabolites of interest. Psilotin is likely stored in the vacuole, in analogy to other (polar) phenolic glycosides (Wink, 1993), while amentoflavone is an apolar biflavonoid aglycone that was previously found to be associated with cell walls (Gadek et al., 1984). Both locations allow for the sequestration of these bioactive metabolites, thereby protecting cellular metabolism in different subcellular locations (Agapakis et al., 2012). Another advantage of the differential localization of psilotin and amentoflavone could be that greater quantities of these pigments can be accumulated, but this hypothesis remains to be tested.

## Data Availability

All datasets generated for this study are included in the manuscript and/or the Supplementary Files.

## Author Contributions

BL conceived the work and wrote the manuscript, with input from all authors. DŠ, VP, NS, and BL designed the experiments and analyzed the data. MW served as mentor for VP. VP generated GC-MS data. DŠ obtained HPLC-QTOF-MS and MALDI-MS data. NS produced and interpreted NMR data and contributed to the generation of new spectral records for the Spektraris online resource.

## Conflict of Interest Statement

The authors declare that the research was conducted in the absence of any commercial or financial relationships that could be construed as a potential conflict of interest.

## Footnotes

1 https://www.r-project.org/

2 https://massbank.eu/MassBank/

3 https://metlin.scripps.edu/

4 https://chemdata.nist.gov/

5 http://langelabtools.wsu.edu/spektraris/

## Supplementary Material

FIGURE S1 Representative GC-MS and HPLC-QTOF-MS chromatograms (total ion current) of *P. nudum* extracts.

FIGURE S2 Absorbance spectra of peaks detected in HPLC-QTOF-MS runs and comparison with those of authentic standards.

FIGURE S3 MS/MS spectra (acquired at 30 eV collision energy) of psilotin and psilotinin.

TABLE S1 Peak identification in GC-MS runs.

TABLE S2 Normalized peak areas for GC-MS and HPLC-QTOF-MS data.

TABLE S3 Component loadings of a PCA analysis with combined GC-MS and HPLC-QTOF-MS data.

TABLE S4 NMR acquisition parameters.

TABLE S5 Bioflavonoids and related metabolites for which new Spektraris spectral records were generated.

## Abbreviations

AMT tag: accurate mass-time tag
CHCA: α-cyano-4-hydroxycinnamic acid
DBA: 2,5-dihydroxybenzoic acid
GC–MS: gas chromatography – mass spectrometry
HPLC–QTOF–MS: high performance liquid chromatography – quadrupole time of flight – mass spectrometry
MALDI–MS: matrix-assisted laser desorption/ionization – mass spectrometry
NMR: nuclear magnetic resonance
PC: principal component
PCA: principal component analysis

## References

[B1] Abul Y. Menéndez V. Gómez-Campo C. Revilla M. A. Lafont F. Fernández H. (2010). Occurrence of plant growth regulators in *Psilotum nudum*. *J. Plant Physiol.* 167 1211–1213. 10.1016/j.jplph.2010.03.015 20488581

[B2] Agapakis C. M. Boyle P. M. Silver P. A. (2012). Natural strategies for the spatial optimization of metabolism in synthetic biology. *Nat. Chem. Biol.* 8 527–535. 10.1038/nchembio.975 22596204

[B3] Akihisa T. Kawashima T. Takahashi S. Sahashi N. Okamoto T. Niiya I. (1992). Sterols and fatty acids of a whisk fern *Psilotum nudum*. *J. Am. Oil Chem. Soc.* 69 1232–1235. 10.1007/BF02637687

[B4] Arnason J. T. Philogène J. R. Donskov N. Muir A. Towers G. H. N. (1986). Psilotin, an insect feeding deterrent and growth reducer from *Psilotum nudum*. *Biochem. Syst. Ecol.* 14 287–289. 10.1016/0305-1978(86)90103-1

[B5] Balza F. Muir A. D. Towers G. H. N. (1985). 3’-Hydroxypsilotin, a minor arylpyrone glycoside from *Psilotum nudum*. *Phytochemistry* 24 529–531. 10.1016/S0031-9422(00)80761-X

[B6] Banks H. P. (1975). Reclassification of the psilophyta. *Taxon* 24 401–413.

[B7] Beck S. Stengel J. (2016). Mass spectrometric imaging of flavonoid glycosides and biflavonoids in Ginkgo biloba L. *Phytochemistry* 130 201–206. 10.1016/j.phytochem.2016.05.005 27233155

[B8] Bino R. J. Hall R. D. Fiehn O. Kopka J. Saito K. Draper J. (2004). Potential of metabolomics as a functional genomics tool. *Trends Plant Sci.* 9 418–425. 10.1016/j.tplants.2004.07.004 15337491

[B9] Chiu P. L. Patterson G. W. Salt T. A. (1988). Sterol composition of pteridophytes. *Phytochemistry* 27 819–822. 10.1016/0031-9422(88)84099-8

[B10] Cooper-Driver G. (1977). Chemical evidence for separating the Psilotaceae from the Filicales. *Science* 198 1260–1262. 10.1126/science.198.4323.1260 17741707

[B11] Cuthbertson D. Andrews P. K. Reganold J. P. Davies N. M. Lange B. M. (2013). Utility of metabolomics toward assessing the metabolic basis of quality traits in apple fruit with an emphasis on antioxidants. *J. Agric. Food Chem.* 60 8552–8560. 10.1021/jf3031088 22881116 PMC3551554

[B12] Doyle J. A. (2018). Phylogenetic analyses and morphological innovations in land plants. *Annu. Plant Rev.* 45 1–50. 10.1002/9781119312994.apr0486

[B13] Ducket J. G. Ligrone R. (2005). A comparative cytological analysis of fungal endophytes in the sporophyte rhizomes and vascularized gametophytes of Tmesipteris and Psilotum. *Can. J. Bot.* 83 1443–1456. 10.1139/b05-102

[B14] Feuereisen M. M. Gamero Barraza M. Zimmermann B. F. Schieber A. Schulze-Kaysers N. (2017). Pressurized liquid extraction of anthocyanins and biflavonoids from *Schinus terebinthifolius* Raddi: a multivariate optimization. *Food Chem.* 214 564–571. 10.1016/j.foodchem.2016.07.002 27507511

[B15] Fiehn O. Sumner L. W. Rhee S. Y. Ward J. Dickerson J. Lange B. M. (2007). Minimum reporting standards for plant biology context information in metabolomic studies. *Metabolomics* 3 195–201. 10.1007/s11306-007-0068-0

[B16] Fischedick J. T. Johnson S. R. Ketchum R. E. Croteau R. B. Lange B. M. (2015). NMR spectroscopic search module for Spektraris, an online resource for plant natural product identification taxane diterpenoids from Taxus × media cell suspension cultures as a case study. *Phytochemistry* 113 87–95. 10.1016/j.phytochem.2014.11.020 25534952 PMC4441555

[B17] Gadek P. A. Quinn C. J. Ashford A. E. (1984). Loclization of the biflavonoid fraction in plant leaves, with special reference to Agathis robusta (C. *Moore Ex F. Muell.) F.m. Bail*. *Austr. J. Bot.* 32 15–31. 10.1071/BT9840015

[B18] Geiger H. Quinn C. (1988). “Biflavonoids,” in The Flavonoids. Advances in Research Since 1980 ed. Harborne J. B. (London: Chapman and Hall), 99–124. 10.1007/978-1-4899-2913-6_4

[B19] Geiger H. Seeger T. Hahn H. Zinsmeister D. Markham K. Wong H. (1993). 1H NMR assignments in biflavonoid spectra by proton-detected C-H correlation. *Z. Naturforsch.* 48c, 821–826. 10.1515/znc-1993-11-1201

[B20] Horai H. Arita M. Kanaya S. Nihei Y. Ikeda T. Suwa K. (2010). MassBank: a public repository for sharing mass spectral data for life sciences. *J. Mass Spectrom.* 45 703–714. 10.1002/jms.1777 20623627

[B21] Iwashina T. (2000). The structure and distribution of the flavonoids in plants. *J. Plant Sci.* 113 287–299. 10.1007/PL00013940

[B22] Kachlicki P. Piasecka A. Stobiecki M. Marczak Ł (2016). Structural characterization of flavonoid glycoconjugates and their derivatives with mass spectrometric techniques. *Molecules* 21:1494. 10.3390/molecules21111494 27834838 PMC6273528

[B23] Kenrick P. Crane P. R. (1997). The origin and early evolution of plants on land. *Nature* 389 33–39. 10.1038/37918

[B24] Lange B. M. Fischedick J. T. Lange M. F. Srividya N. Šamec D. Poirier B. C. (2017). Integrative approaches for the identification and localization of specialized metabolites in Tripterygium roots. *Plant Physiol.* 173 456–469. 10.1104/pp.15.01593 27864443 PMC5210757

[B25] Markham K. R. (1984). The structures of amentoflavone glycosides isolated from *Psilotum nudum*. *Phytochemistry* 23 2053–2056. 10.1016/S0031-9422(00)84969-9

[B26] McInnes A. G. Yoshida S. Towers G. H. N. (1965). A arylpyrone glycoside from *Psilotum nudum* (L) Griseb. *Tetrahedron* 21 2939–2946. 10.1016/S0040-4020(01)98380-2

[B27] Nilsen E. T. (1995). “Stem photosynthesis: extent, patterns, and role in plant carbon economy,” in *Plant Above-Ground Rhizomes: Physiology and Functional Morphology*, ed. Gartner B. (San Diego: Academic Press), 223–240. 10.1016/b978-012276460-8/50012-6

[B28] Pittermann J. Limm E. Rico C. Christman M. A. (2011). Structure-function constraints of tracheid-based xylem: a comparison of conifers and ferns. *New Phytol.* 192 449–461. 10.1111/j.1469-8137.2011.03817.x 21749396

[B29] Pryer K. M. Schneider H. Smith A. R. Cranfill R. Wolf P. G. Hunt J. S. (2001). Horsetails and ferns are a monophyletic group and the closest living relatives to seed plants. *Nature* 409 618–622. 10.1038/35054555 11214320

[B30] Renzaglia K. S. Johnson T. H. Gates H. D. Whittier D. P. (2001). Architecture of the sperm cell of Psilotum. *Am. J. Bot.* 88 1151–1163. 10.2307/3558326 11454615

[B31] Rothwell G. W. Millay M. A. Stockey R. A. (2018). Resolving the overall pattern of marattialean fern phylogeny. *Am. J. Bot.* 105 1304–1314. 10.1002/ajb2.1115 30001474

[B32] Shamsuddin T. Khan S. A. Ahmad I. Rahman W. Shamsuddin K. M. (1985). Psilotic acid, a C6-C4-acid from *Psilotum nudum*. *Phytochemistry* 24 2458–2459. 10.1016/S0031-9422(00)83070-8

[B33] Siegel S. M. (1976). Inhibitory activity of the arylpyrone glucoside psilotin and its reversal by gibberellic acid and thiols. *Phytochemistry* 15 566–567. 10.1016/S0031-9422(00)88980-3

[B34] Takahashi M. Yamane H. Satoh Y. Takahashi N. Iwatsuki K. (1984). Identification of GA36 in *Psilotum nudum*. *Phytochemistry* 23:681 10.1016/S0031-9422(00)80407-0

[B35] Takahashi S. Nakamura F. Sahashi N. Ohmoto T. Mizushima U. Sankawa U. (1990). Chemical markers of the Psilotaceae. *Biochem. Syst. Ecol.* 18 11–12. 10.1016/0305-1978(90)90024-A

[B36] Tse A. Towers G. H. N. (1967). The occurrence of psilotin in Tmesipteris. *Phytochemistry* 6:149. 10.1016/0031-9422(67)85023-4 8635473

[B37] Wallace J. W. Markham K. R. (1978). Apigenin and amentoflavone glycosides in the psilotaceae and their phylogenetic significance. *Phytochemistry* 17 1313–1317. 10.1016/S0031-9422(00)94580-1

[B38] Wang G. Yao S. Zhang X. X. Song H. (2015). Rapid screening and structural characterization of antioxidants from the extract of Selaginella doederleinii Hieron with DPPH-UPLC-Q-TOF/MS method. *Int. J. Anal. Chem* 2015:849769. 10.1155/2015/849769 25792983 PMC4352518

[B39] Waterman M. J. Nugraha A. S. Hendra R. Ball G. E. Robinson S. A. Keller P. A. (2017). Antarctic moss biflavonoids show high antioxidant and ultraviolet-screening activity. *J. Nat. Prod.* 80 2224–2231. 10.1021/acs.jnatprod.7b00085 28783339

[B40] White R. A. Turner M. D. (1995). Anatomy and development of the fern sporophyte. *Bot. Rev.* 61 281–305. 10.1007/BF02912620

[B41] Wink M. (1993). The plant vacuole: a multifunctional compartment. *J. Exp. Bot.* 44 231–246.

[B42] Yamaguchi L. F. Kato M. J. Di Mascio P. (2009). Biflavonoids from Araucaria angustifolia protect against DNA UV-induced damage. *Phytochemistry* 70 615–620. 10.1016/j.phytochem.2009.03.003 19349053

[B43] Yu S. Yan H. Zhang L. Shan M. Chen P. Ding A. (2017). A review on the phytochemistry, pharmacology, and pharmacokinetics of amentoflavone, a naturally-occurring biflavonoid. *Molecules* 22:299. 10.3390/molecules22020299 28212342 PMC6155574

[B44] Yu X. Wang J. Zhang J. Wang L. Wang Z. Xiong F. (2015). Physicochemical properties of starch isolated from bracken (Pteridium aquilinum) rhizome. *J. Food Sci.* 80 C2717–C2724. 10.1111/1750-3841.13129 26551243

[B45] Zhang Y. X. Li Q. Y. Yan L. L. Shi Y. (2011). Structural characterization and identification of biflavones in Selaginella tamariscina by liquid chromatography-diode-array detection/electrospray ionization tandem mass spectrometry. *Rapid Commun. Mass Spectrom.* 25 2173–2186. 10.1002/rcm.5090 21710597

[B46] Zhang S. Zhong G. Liu B. Wang B. (2011). Physicochemical and functional properties of fern rhizome (Pteridium aquilinum) starch. *Starch/Stärke* 63 468–474. 10.1002/star.201000142 26551243

