# Supplementary material for: Assessing Chemical Diversity in Psilotum nudum (L.) Beauv., a Pantropical Whisk Fern That Has Lost Many of Its Fern-Like Characters
Source: Front Plant Sci. 2019 Jul 9;10:868. doi: 10.3389/fpls.2019.00868 (PMC6629931; doi:10.3389/fpls.2019.00868)

Supplementary Figure 1 | Representative GC-MS and HPLC-QTOF-MS chromatograms (total ion current) of *P. nudum* extracts.

Samec et al. (2019) Assessing Chemical Diversity in *Psilotum nudum* (L.) Beauv., a Pantropical Whisk Fern That Has Lost Many of its Fern-like Characters. *Frontiers in Plant Science*

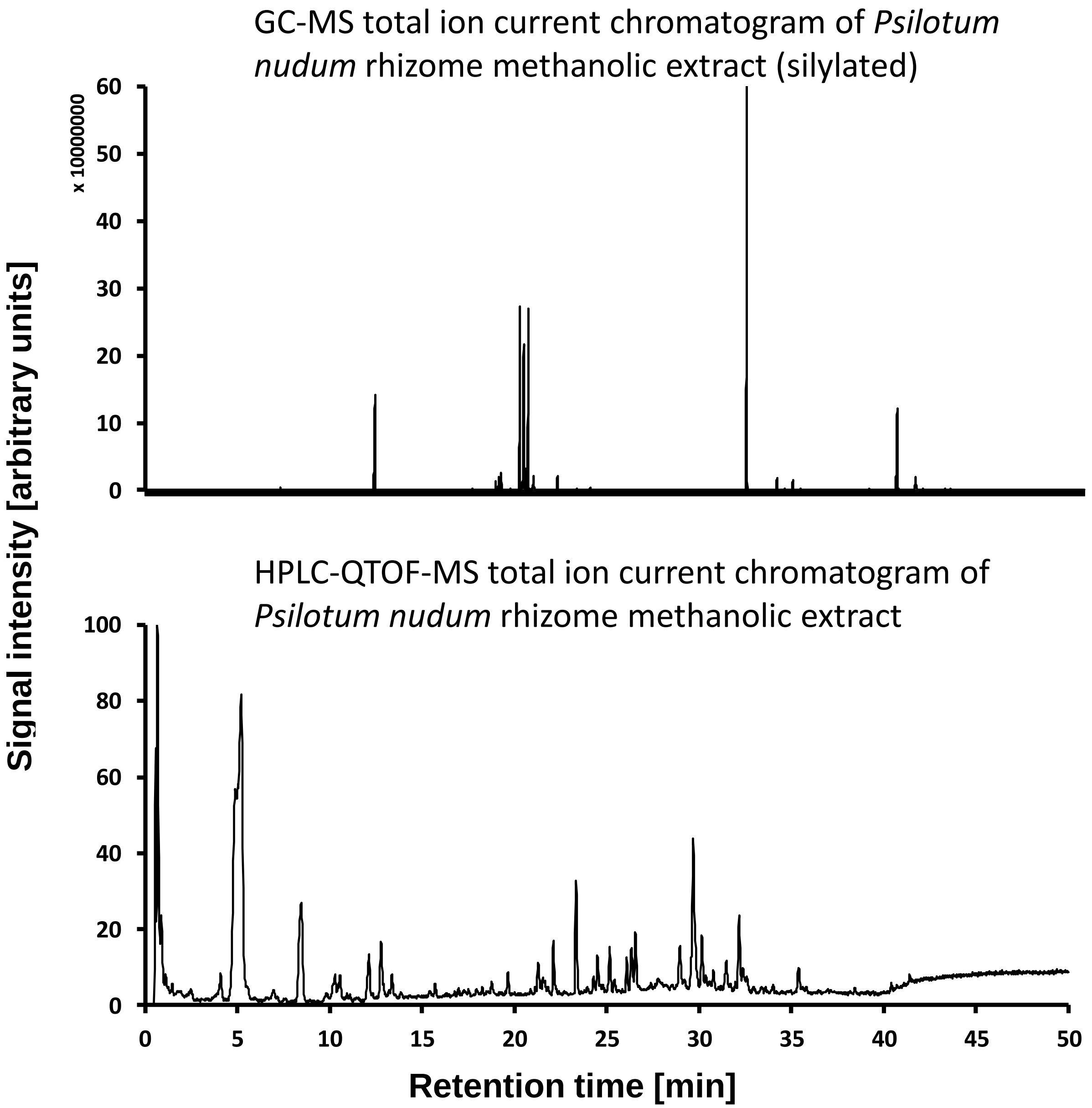

Supplement: FIGURE S1 — Representative GC-MS and HPLC-QTOF-MS chromatograms (total ion current) of P. nudum extracts. [file Image_1.pdf]
