# Supplementary material for: Assessing Chemical Diversity in Psilotum nudum (L.) Beauv., a Pantropical Whisk Fern That Has Lost Many of Its Fern-Like Characters
Source: Front Plant Sci. 2019 Jul 9;10:868. doi: 10.3389/fpls.2019.00868 (PMC6629931; doi:10.3389/fpls.2019.00868)

Supplementary Figure 2 | Absorbance spectra of peaks detected in HPLC-QTOF-MS runs and comparison with those of authentic standards.

Samec et al. (2019) Assessing Chemical Diversity in *Psilotum nudum* (L.) Beauv., a Pantropical Whisk Fern That Has Lost Many of its Fern-like Characters. *Frontiers in Plant Science*

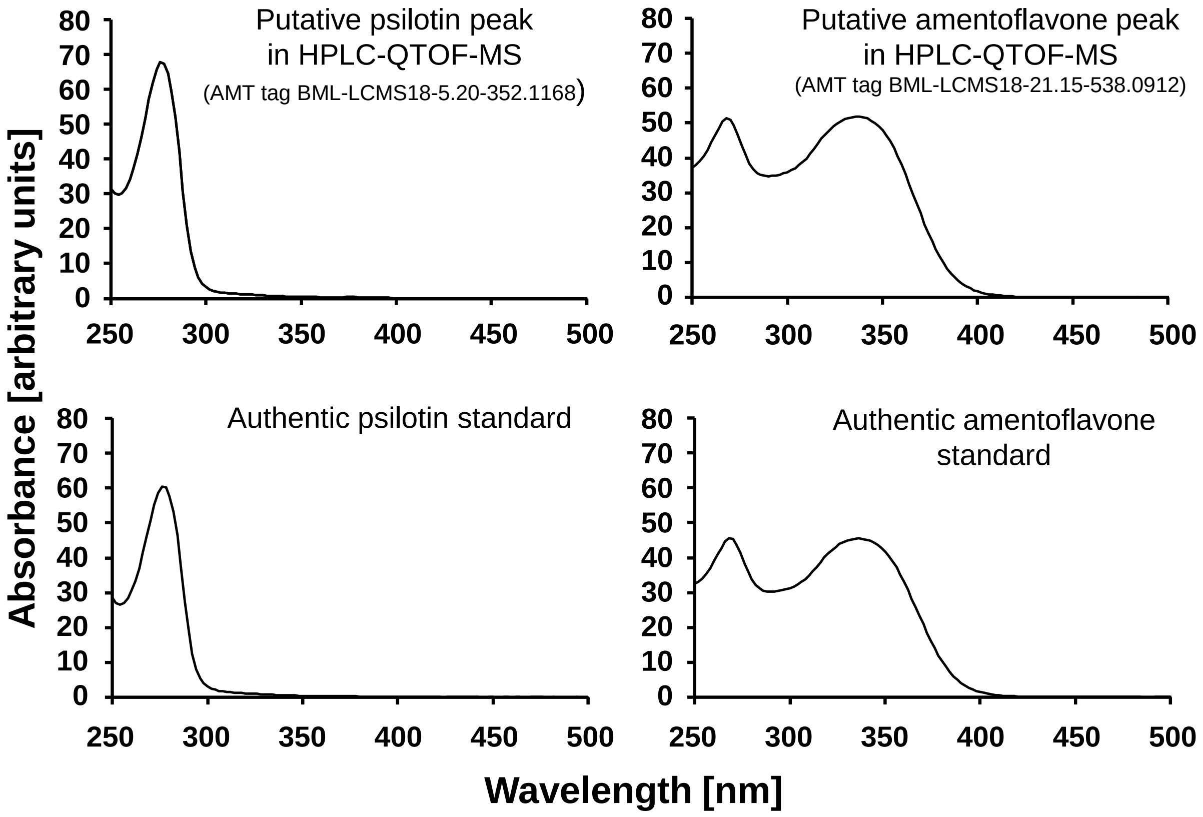

Supplement: FIGURE S2 — Absorbance spectra of peaks detected in HPLC-QTOF-MS runs and comparison with those of authentic standards. [file Image_2.pdf]
