# Supplementary material for: Assessing Chemical Diversity in Psilotum nudum (L.) Beauv., a Pantropical Whisk Fern That Has Lost Many of Its Fern-Like Characters
Source: Front Plant Sci. 2019 Jul 9;10:868. doi: 10.3389/fpls.2019.00868 (PMC6629931; doi:10.3389/fpls.2019.00868)

Supplementary Figure 3 | MS/MS spectra (acquired at 30 eV collision energy) of psilotin and psilotinin.

Samec et al. (2019) Assessing Chemical Diversity in *Psilotum nudum* (L.) Beauv., a Pantropical Whisk Fern That Has Lost Many of its Fern-like Characters. *Frontiers in Plant Science*

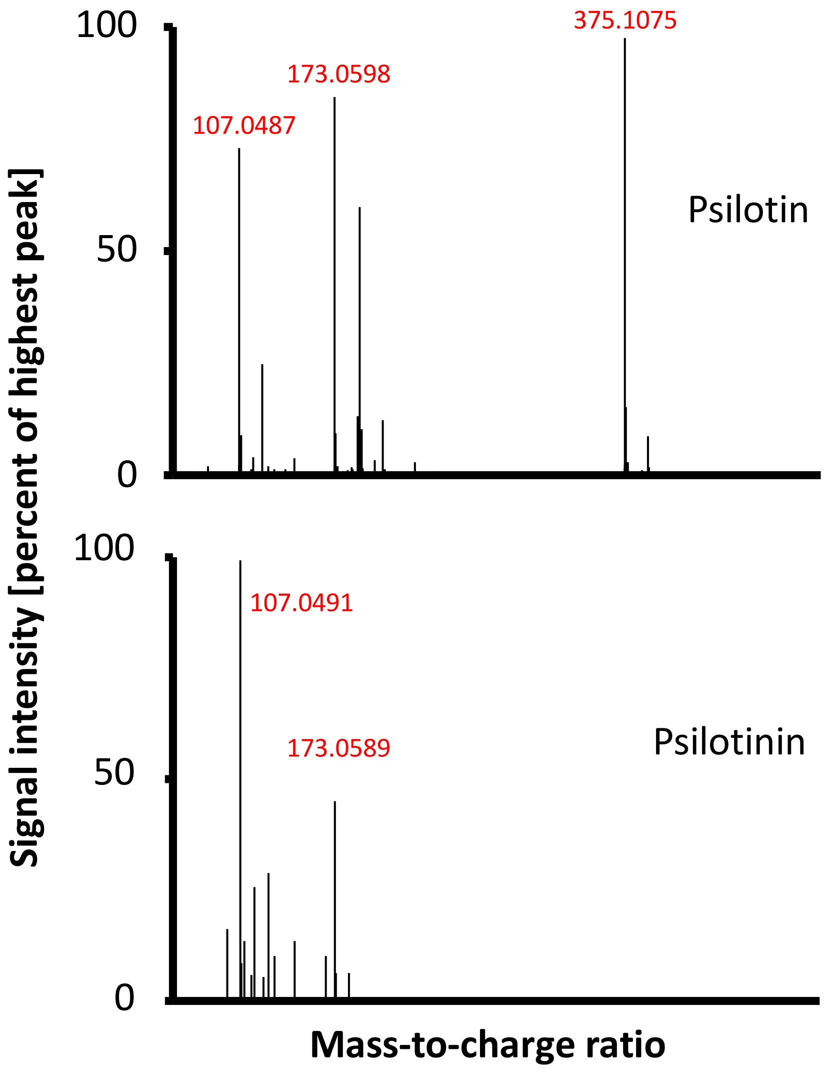

Supplement: FIGURE S3 — MS/MS spectra (acquired at 30 eV collision energy) of psilotin and psilotinin. [file Image_3.pdf]
